# Activation of Autophagy Relieves Linoleic Acid-Induced Inflammation in Large Yellow Croaker (*Larimichthys crocea*)

**DOI:** 10.3389/fimmu.2021.649385

**Published:** 2021-06-30

**Authors:** Bo Yang, Renlei Ji, Xueshan Li, Wei Fang, Qiuchi Chen, Qiang Chen, Wei Xu, Kangsen Mai, Qinghui Ai

**Affiliations:** ^1^ Key Laboratory of Aquaculture Nutrition and Feed (Ministry of Agriculture and Rural Affairs) & Key Laboratory of Mariculture (Ministry of Education), Ocean University of China, Qingdao, China; ^2^ Laboratory for Marine Fisheries Science and Food Production Processes, Qingdao National Laboratory for Marine Science and Technology, Qingdao, China

**Keywords:** autophagy, inflammation, linoleic acid, large yellow croaker, *in vivo*, *in vitro*

## Abstract

High levels of soybean oil (SO) in fish diets enriched with linoleic acid (LA, 18:2n-6) could induce strong inflammation. However, the molecular mechanism underlying LA-induced inflammation in the liver of large yellow croaker (*Larimichthys crocea*) has not been elucidated. Based on previous research, autophagy has been considered a new pathway to relieve inflammation. Therefore, the present study was performed to investigate the role of autophagy in regulating LA-induced inflammation in the liver of large yellow croaker *in vivo* and *in vitro.* The results of the present study showed that activation of autophagy in liver or hepatocytes could significantly reduce the gene expression of proinflammatory factors, such as tumor necrosis factor α (TNFα) and interleukin 1β (IL1β). The results of the present study also showed that inhibition of autophagy could upregulate the gene expression of proinflammatory factors and downregulate the gene expression of anti-inflammatory factors *in vivo* and *in vitro*. Furthermore, autophagy could alleviate LA-induced inflammatory cytokine gene expression *in vivo* and *in vitro*, while inhibition of autophagy obtained the opposite results. In conclusion, our study shows that autophagy could regulate inflammation and alleviate LA-induced inflammation in the liver of large yellow croaker *in vivo* and *in vitro* for the first time, which may offer considerable benefits to the aquaculture industry and human health.

## Introduction

Fish oil (FO) contains a relatively high content of long-chain polyunsaturated fatty acids (LC-PUFAs) and is the major lipid component of traditional fish diets. However, increasing demand, uncertain availability and high price of fish oil have necessitated the search for alternative lipid sources (1). Soybean oil has abundant linoleic acid content, considerable output and an acceptable price and thus represents a promising alternative to fish oil. However, numerous studies have demonstrated that high linoleic acid levels in diets could lead to a decrease in growth, antioxidant capacity and immunity in large yellow croaker (2, 3). Many studies on the nutritional immunity of large yellow croaker have been conducted in recent years (4). However, no information is available on the molecular solution of linoleic acid-induced inflammation.

Although LA causes strong inflammation, fish can generally maintain normal growth and immune responses; thus, protection and mitigation mechanisms must be utilized. Based on previous research, autophagy is a general term for pathways for the delivery of cytoplasmic material, including soluble macromolecules and organelles, to lysosomes for degradation (5, 6). In mammals, autophagy is associated with many pathological processes and considered a new pathway to relieve inflammation (7). Studies have revealed that autophagy plays a critical role in eliminating damaged organelles, resisting oxidase stress and energy deficiency, and controlling innate and adaptive immune responses, which partly occurs through regulating inflammatory cytokine production (8–10). In recent years, autophagy has been found to be involved in the fish immune defense system. Wang et al. verified that autophagy inhibited the replication of snakehead fish vesiculovirus (SHVV) in a snakehead fish cell line (SSN-1) (11). Li et al. found that autophagy promoted infectious kidney and spleen necrosis virus (ISKNV) replication and decreased infectious virus yields in a Chinese perch brain cell line (CPB cell line) (12). Although the roles of autophagy have been studied in many model organisms, the roles of autophagy in inflammation within large yellow croaker and its roles in regulating linoleic acid-induced inflammation in the liver of large yellow croaker have not been fully elucidated.

Large yellow croaker is one of the most commercially important aquacultural fish species in China. Our previous studies indicated that inflammation of large yellow croaker is strikingly similar to that of other fish species and mammals, which suggests that large yellow croaker may be a useful model for studying inflammation (13). Thus, in this study, the role of autophagy in regulating LA-induced inflammation in the liver of large yellow croaker was studied *in vivo* and *in vitro*.

## Materials and Methods

### Injection Experiments

Large yellow croaker were raised in floating sea cages (4×4×2.5 m) and acclimated to the experimental environments and diets before the start of the injection experiment (**Supplementary Tables 1**–**3**) (14). After fasting for 24 h, the fish were randomly divided into 36 floating cages with 30 fish per cage (1×1×2 m). For experiment one, the fish were intraperitoneally injected with increasing doses of rapamycin (RAPA, Sigma-Aldrich, St. Louis, MO, USA) (0.0 mg/kg, 0.5 mg/kg, 1.0 mg/kg, 1.5 mg/kg, 2 mg/kg and 2.5 mg/kg) or chloroquine (CQ, MCE, Shanghai, China) (0.0 mg/kg, 10 mg/kg, 20 mg/kg, 30 mg/kg, 40 mg/kg and 50 mg/kg). Each treatment was randomly assigned to triplicate cages. The liver tissues were sampled after injection with RAPA for 12 h and 48 h or after injection with CQ for 24 h and 48 h. For experiment two, after the 70-day soybean oil diet feeding experiment, the large yellow croaker in the original net cage was anaesthetized and divided into 4 net cages (1×1×2 m). Then, the fish were intraperitoneally injected with PBS (Biological Industries, Beit Haemek, Israel), 1.0 mg/kg RAPA or 40 mg/kg CQ for 48 h. Injection with PBS was used as a control group.

### Primary Hepatocyte Isolation and Culture

Large yellow croakers were treated in seawater containing 1000 IU/mL penicillin (Sangon Biotech, China) and 1.2 mg/mL streptomycin (Sangon Biotech, China) for 24 h. The livers were dissected under sterile conditions and collected in cold sterile PBS supplemented with 100 U/ml penicillin and 100 μg/ml streptomycin. After washing with Dulbecco’s modified Eagle’s medium (DMEM/F12) (Biological Industries, Beit Haemek, Israel), liver tissue was minced into 1 mm^3^ pieces and digested with 0.25% trypsin-EDTA (Gibco, USA) at room temperature for 10 min. The reaction was terminated by adding DMEM/F12 medium containing 15% fetal bovine serum (FBS), and the cell suspension was purified through a cell strainer with 70 μm mesh. The isolated cells were collected in a 15 ml sterile centrifuge tube and centrifuged at 500 g for 10 min at 4°C. Cell pellets were resuspended in complete medium (DMEM/F12 medium containing 15% FBS, 100 U/ml penicillin and 100 μg/ml streptomycin). Primary hepatocytes were seeded into six-well culture dishes at a density of 2×10^6^ cells/well and incubated at 28°C in 5% CO_2_.

### Cell Treatment

Primary hepatocytes were serum-starved with FBS-free DMEM/F12 for 2 h prior to the experiment. For experiment one, primary hepatocytes were incubated in 1 mM linoleic acid conjugated with fatty acid-free bovine serum albumin (BSA) in DMEM/F12 for 12 h. For experiment two, primary hepatocytes were treated with increasing doses of autophagy activator RAPA (0 nM, 100 nM, 200 nM, 300 nM, 400 nM and 500 nM) for 6 h or autophagy inhibitor CQ (0 µM, 1 µM, 2 µM, 5 µM, 10 µM and 20 µM) for 6 h. For experiment three, primary hepatocytes were treated with 200 nM RAPA for 6 h and 12 h or 5 µM CQ for 6 h and 12 h. For experiment four, primary hepatocytes were pretreated with 1 mM LA for 12 h. Then, the medium was replaced with fresh medium supplemented with 200 nM RAPA for 12 h or 5 µM CQ for 12 h. Cells were harvested after treatment for the indicated times. All cell culture experiments were repeated at least three times.

### Real-Time Polymerase Chain Reaction (qRT-PCR)

Real-time polymerase chain reaction was performed as previously described (15). Three replicate extractions were performed for each sample. The primers were designed following the published sequences (**Table 1**). The housekeeping genes, GAPDH and β-actin were chosen for the reference genes. To calculate the expression of immune-related genes, the comparative CT method (2^-△△CT^ method) was adopted, and the value represents the n-fold difference relative to the calibration.

**Table 1:** Primers used in the present study.

Primer names	Forward primer sequence (5’ to 3’)
MyD88-F/R	TACGAAGCGACCAATAACCC/ATCAATCAAAGGCCGAAGAT
Cox-2-F/R	ACCATCTGGCTGCGGGAAC/GAATGAGTCGTGTGGTCTGGAAG
IL6-F/R	GCTGCTGAGGAACATCGACAC/GCTGCTCCCATTTTCTGAACT
IFNγ-F/R	AAAGCATTGTGGGAGCTGTCG/CCTCTTAGGTGGTTTCTGTTCG
Arg I-F/R	AACCACCCGCAGGATTACG/AAACTCACTGGCATCACCTCA
IL10-F/R	AGTCGGTTACTTTCTGTGGTG/TGTATGACGCAATATGGTCTG
IL1β-F/R	CATCTGGAGGCGGTGGAGGA/GGGACAGACCTGAGGGTGGT
TNFα-F/R	CGTCGTTCAGAGTCTCCTGC/TGTACCACCCGTGTCCCACT
βactin-F/R	GACCTGACAGACTACCTCATG/AGTTGAAGGTGGTCTCGTGGA

MyD88, myeloid differentiation factor 88; Cox-2, cyclo-oxygenase-2; IL6, interleukin 6; IFNγ, interferon gamma; Arg I, arginase I; IL10, interleukin 10; IL1β, interleukin 1β; TNFα, tumour necrosis factor α; βactin, Beta-actin; F, forward; R, reverse.

### Western Blot

Total liver proteins were extracted according to previously described methods (16). The protein content was quantified using a Bradford Protein Assay Kit (Beyotime Institute of Technology, China). An equal amount of protein (20 µg) was loaded into wells and separated by 15% sodium dodecyl sulfate polyacrylamide gel electrophoresis. After electrophoresis, proteins in the gel were transferred to a polyvinylidene fluoride (PVDF) membrane (Millipore, USA), followed by membrane blocking at room temperature for 2 h. The PVDF membranes were incubated with primary antibody overnight in a freezer. The membranes were then washed five times for 3 min each with Tris-buffered saline with Tween (TBST) and incubated for 2 h with horseradish peroxide (HRP)-conjugated secondary antibody in TBST. Immune complexes were visualized using an electrochemiluminescence (ECL) kit (Beyotime Institute of Technology, China). Polyclonal anti-LC3 (cat. no. 7543) was obtained from Sigma-Aldrich (USA). Anti-glyceraldehyde 3-phosphate dehydrogenase (GAPDH) (cat. no. 309154) and HRP-conjugated secondary antibodies (cat. no. ZB-2301) were obtained from Golden Bridge Biotechnology (China).

### Statistical Analysis

Statistical analysis was performed in SPSS 20.0 (SPSS Inc., USA). Data from each treatment were subjected to one-way analysis of variance (ANOVA) followed by independent samples T-test and Tukey’s multiple-range test. Statistically significant differences were identified at *P* < 0.05. The results are presented as the mean ± SEM (standard error of the mean).

## Results

### Activation of Autophagy Could Down-Regulate the Inflammation Level *In Vivo*

**Figure 1:**
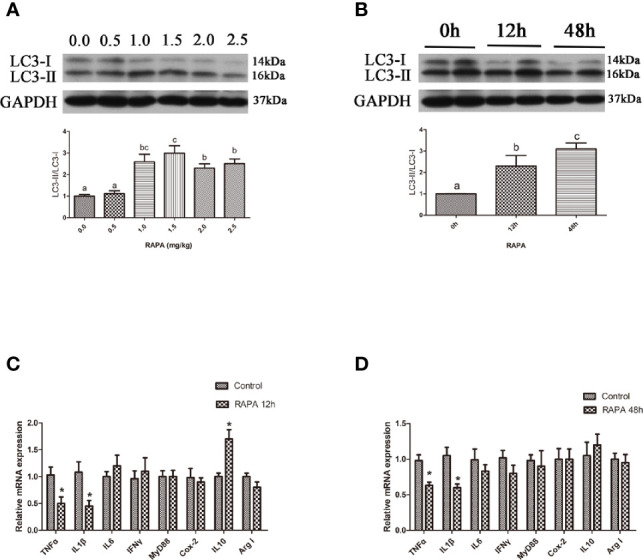
Activation of autophagy could down-regulate the expression of inflammatory genes *in vivo*. **(A)** The ratio of LC3-II/LC3-I was analyzed by western blots on liver after treatment of different levels of RAPA (n=3). **(B)** The ratio of LC3-II/LC3-I was analyzed by western blots on liver after treatment of different time of RAPA (n=3). **(C)** The mRNA expressions of TNFα, IL1β, IL6, IFNγ, MyD88, Cox-2, IL10 and Arg I were analyzed by qRT-PCR on liver after injected with 1.0mg/kg RAPA for 12h (n=6). **(D)** The mRNA expressions of TNFα, IL1β, IL6, IFNγ, MyD88, Cox-2, IL10 and Arg I were analyzed by qRT-PCR on liver after injected with 1.0mg/kg RAPA for 48h (n=6). Results were represented as means with SEM and significance was evaluated by one-way ANOVA followed by Tukey’s multiple range tests or independent *t* tests (*P < 0.05). ^a,b,c^Means share a same superscript letter are not significantly different (*P* ≥ 0.05).

Rapamycin, a mammalian target of rapamycin (mTOR) inhibitor (17), is a well-established autophagy activator. Our results showed that the ratio of LC3-II/LC3-I in the liver significantly increased with increasing levels of RAPA injection from 1.0 mg/kg to 2.5 mg/kg (*P* < 0.05) (**Figures 1A, B**). Moreover, RAPA injection also affected the expression of inflammatory genes. More specifically, compared with the control group, 1.0 mg/kg RAPA injection for 12 h significantly increased the transcript expression levels of the anti-inflammatory cytokine IL10 and significantly decreased the transcript expression levels of the proinflammatory cytokines TNFα and IL1β (*P* < 0.05) (**Figure 1C**); in addition, 1.0 mg/kg RAPA injection for 48 h significantly decreased the transcript expression levels of the proinflammatory cytokines TNFα and IL1β (*P* < 0.05) (**Figure 1D**). These results indicate that activation of autophagy could downregulate the inflammation level *in vivo*.

### Activation of Autophagy Could Downregulate the Inflammation Level *In Vitro*

**Figure 2:**
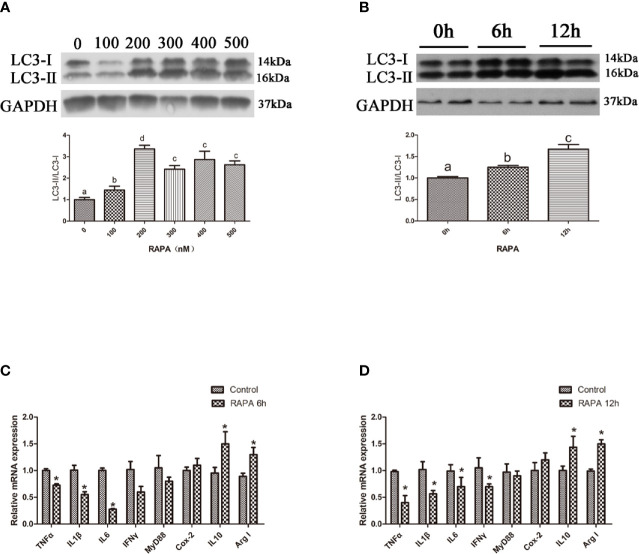
Activation of autophagy could down-regulate the expression of inflammatory genes *in vitro*. **(A)** The ratio of LC3-II/LC3-I was analyzed by western blots in primary hepatocyte after treatment of different levels of RAPA (n=3). **(B)** The ratio of LC3-II/LC3-I was analyzed by western blots in primary hepatocyte after treatment of different time of RAPA (n=3). **(C)** The mRNA expressions of TNFα, IL1β, IL6, IFNγ, MyD88, Cox-2, IL10 and Arg I were analyzed by qRT-PCR in primary hepatocyte after treatment of 200 nM RAPA for 6h (n=6). **(D)** The mRNA expressions of TNFα, IL1β, IL6, IFNγ, MyD88, Cox-2, IL10 and Arg I were analyzed by qRT-PCR in primary hepatocyte after treatment of 200 nM RAPA for 12h (n=6). Results were represented as means with SEM and significance was evaluated by one-way ANOVA followed by Tukey’s multiple range tests or independent *t* tests (*P < 0.05). ^a,b,c^Means share a same superscript letter are not significantly different (*P* ≥ 0.05).

In the *in vitro* experiment, the ratio of LC3-II/LC3-I in hepatocytes significantly increased with increasing levels of RAPA from 100 nM to 500 nM (*P* < 0.05) (**Figures 2A, B**). Incubation with 200 nM RAPA for 6 h significantly decreased the transcript expression levels of the proinflammatory cytokines TNFα, IL1β and IL6 and significantly increased the transcript expression levels of the anti-inflammatory cytokines Arg I and IL10 (*P* < 0.05) (**Figure 2C**). Treatment with 200 nM RAPA for 12 h significantly decreased the transcript expression levels of the proinflammatory cytokines TNFα, IL10, IFNγ, and MyD88 and significantly increased the transcript expression levels of anti-inflammatory Arg I and IL10 (*P* < 0.05) (**Figure 2D**). These results indicate that activation of autophagy could downregulate the inflammation level *in vitro*.

### Inhibition of Autophagy Could Upregulate the Inflammation Level *In Vivo*

**Figure 3:**
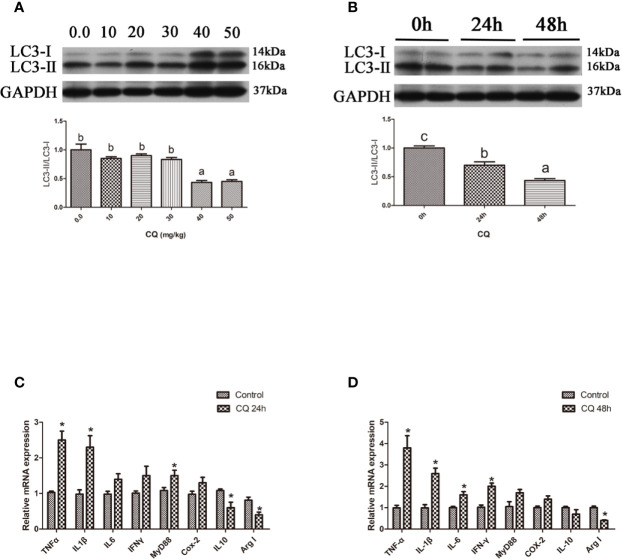
Inhibition of autophagy could up-regulate the expression of inflammatory genes *in vivo*. **(A)** The ratio of LC3-II/LC3-I was analyzed by western blots on liver after treatment of different levels of CQ (n=3). **(B)** The ratio of LC3-II/LC3-I was analyzed by western blots on liver after treatment of different time of CQ (n=3). **(C)** The mRNA expressions of TNFα, IL1β, IL6, IFNγ, MyD88, Cox-2, IL10 and Arg I were analyzed by qRT-PCR on liver after injected with 40 mg/kg CQ for 24h (n=6). **(D)** The mRNA expressions of TNFα, IL1β, IL6, IFNγ, MyD88, Cox-2, IL10 and Arg I were analyzed by qRT-PCR on liver after injected with 40 mg/kg CQ for 48h (n=6). Results were represented as means with SEM and significance was evaluated by one-way ANOVA followed by Tukey’s multiple range tests or independent *t* tests (*P < 0.05). ^a,b,c^Means share a same superscript letter are not significantly different (*P* ≥ 0.05).

Autophagic flux can be blocked by CQ, a lysosome inhibitor (18). Our results showed that the ratio of LC3-II/LC3-I in the liver significantly increased with increasing levels of CQ injection from 40 mg/kg to 50 mg/kg (*P* < 0.05) (**Figures 3A, B**). Compared with the control group, 40 mg/kg CQ injection for 24 h could significantly increase the transcript expression levels of pro-inflammatory cytokines TNFα, IL1β, Myd88 and significantly decrease the transcript expression levels of anti-inflammatory cytokines Arg I and IL10 (*P* < 0.05) (**Figure 3C**), whereas 40 mg/kg CQ injection for 48 h could significantly increase the transcript expression levels of pro-inflammatory cytokines TNFα, IL1β, IL6, IFNγ and significantly decrease the transcript expression levels of anti-inflammatory Arg I (*P* < 0.05) (**Figure 3D**). These results indicate that inhibition of autophagy could upregulate the inflammation level *in vivo*.

### Inhibition of Autophagy Could Upregulate the Inflammation Level *In Vitro*

**Figure 4:**
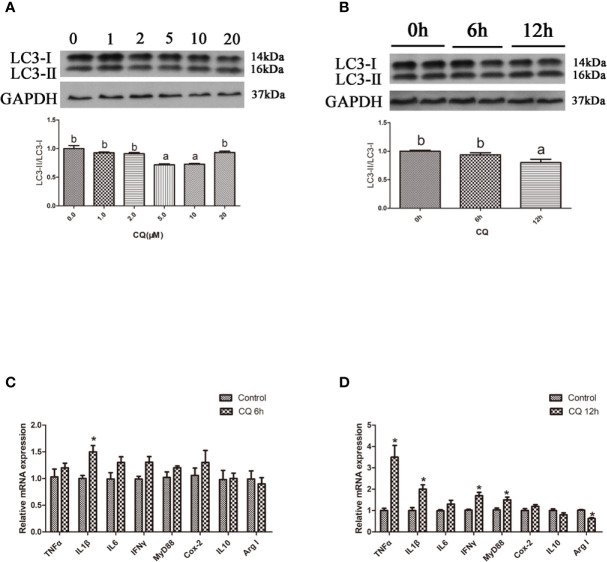
Inhibition of autophagy could up-regulate the expression of inflammatory genes *in vitro*. **(A)** The ratio of LC3-II/LC3-I was analyzed by western blots in primary hepatocyte after treatment of different levels of CQ (n=3). **(B)** The ratio of LC3-II/LC3-I was analyzed by western blots in primary hepatocyte after treatment of different time of CQ (n=3). **(C)** The mRNA expressions of TNFα, IL1β, IL6, IFNγ, MyD88, Cox-2, IL10 and Arg I were analyzed by qRT-PCR in primary hepatocyte after treatment of 5 µM CQ for 6h (n=6). **(D)** The mRNA expressions of TNFα, IL1β, IL6, IFNγ, MyD88, Cox-2, IL10 and Arg I were analyzed by qRT-PCR in primary hepatocyte after treatment of 5 µM CQ for 12h (n=6). Results were represented as means with SEM and significance was evaluated by one-way ANOVA followed by Tukey’s multiple range tests or independent *t* tests (*P < 0.05). ^a,b,c^Means share a same superscript letter are not significantly different (*P* ≥ 0.05).

In the *in vitro* experiment, the ratio of LC3-II/LC3-I in hepatocytes significantly increased with increasing levels of CQ from 5 μM to 10 μM (*P* < 0.05) (**Figures 4A, B**). Incubation with 5 μM CQ for 6 h significantly increased the transcript expression levels of the proinflammatory cytokine TNFα (*P* < 0.05) (**Figure 4C**). After 12 h, incubation with 5 μM CQ significantly increased the transcript expression levels of the proinflammatory cytokines TNFα, IL1β, IFNγ and MyD88 and significantly decreased the transcript expression levels of anti-inflammatory Arg I (*P* < 0.05) (**Figure 4D**). These results indicate that inhibition of autophagy could upregulate the inflammation level *in vitro*.

### Autophagy Could Reduce LA-Induced Inflammatory Cytokine Gene Expression *In Vivo*

**Figure 5:**
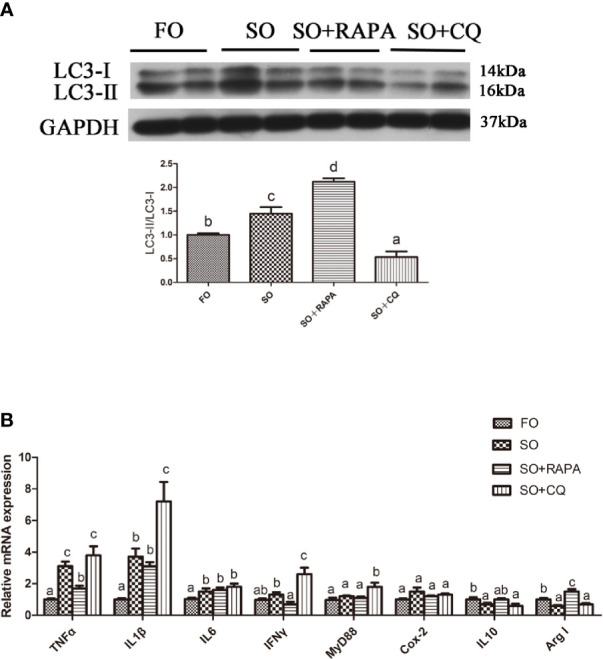
Autophagy could reduce SO-induced inflammatory *in vivo*. **(A)** The ratio of LC3-II/LC3-I was analyzed by western blots on liver after treatment of FO, SO, SO+RAPA and SO+CQ (n=3). FO group as a negative control. **(B)** The mRNA expressions of TNFα, IL1β, IL6, IFNγ, MyD88, Cox-2, IL10 and Arg I were analyzed by qRT-PCR on liver after treatment of FO, SO, SO+RAPA and SO+CQ (n=6). Results were represented as means with SEM and significance was evaluated by one-way ANOVA followed by Tukey’s multiple range tests (*P* < 0.05). ^a,b,c^Means share a same superscript letter are not significantly different (*P* ≥ 0.05).

The gene expression of the proinflammatory cytokines TNFα and IFNγ in the liver of large yellow croaker injected with RAPA was significantly lower than that in croaker fed the SO diet (*P* < 0.05) (**Figures 5A, B**). Compared with that in the SO group, the gene expression of the anti-inflammatory cytokine Arg I in the liver of large yellow croaker injected with RAPA was significantly increased (*P* < 0.05) (**Figure 5B**). CQ injection significantly upregulated SO-induced pro-inflammatory cytokines IL1β, IFNγ and MyD88 mRNA in the liver but did not significantly influence anti-inflammatory cytokine mRNA expression (*P* < 0.05) (**Figure 5B**). These results indicate that autophagy plays a role in regulating LA-induced inflammation *in vivo*.

### Autophagy Could Reduce LA-Induced Inflammatory Cytokine Gene Expression *In Vitro*

**Figure 6:**
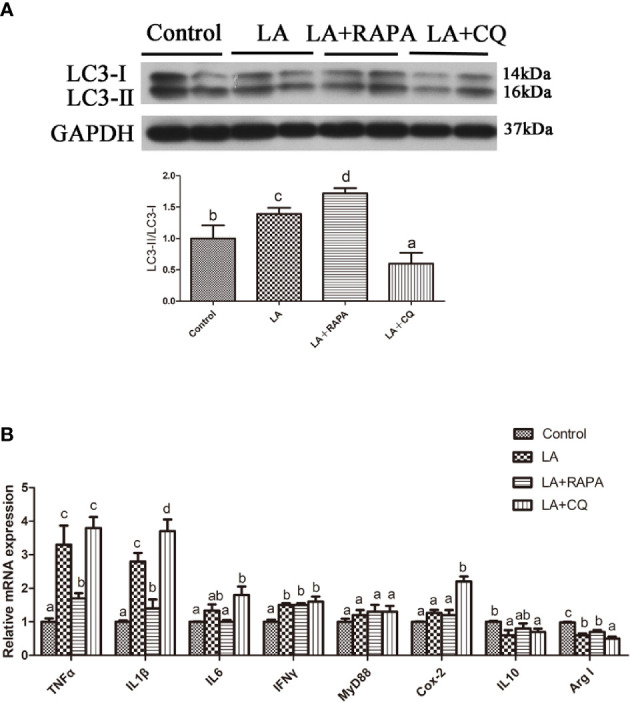
Autophagy could reduce LA-induced inflammatory *in vitro*. **(A)** The ratio of LC3-II/LC3-I was analyzed by western blots in primary hepatocyte after treatment of BSA, LA, LA+RAPA and LA+CQ (n=3). BSA group as a negative control. **(B)** The mRNA expressions of TNFα, IL1β, IL6, IFNγ, MyD88, Cox-2, IL10 and Arg I were analyzed by qRT-PCR in primary hepatocyte after treatment of BSA, LA, LA+RAPA and LA+CQ (n=6). Results were represented as means with SEM and significance was evaluated by one-way ANOVA followed by Tukey’s multiple range tests (*P* < 0.05). ^a,b,c^Means share a same superscript letter are not significantly different (*P* ≥ 0.05).

Pretreatment with RAPA significantly reduced LA-induced mRNA expression of the proinflammatory cytokines TNFα and IL1β in hepatocytes but did not significantly influence mRNA expression of the anti-inflammatory cytokines (*P* < 0.05) (**Figures 6A, B**). The gene expression of the proinflammatory cytokines IL1β and Cox2 in hepatocytes treated with CQ was significantly higher than that in hepatocytes treated with LA (*P* < 0.05) (**Figure 6B**). Compared with that in the LA group, the gene expression of the anti-inflammatory cytokine Arg I in hepatocytes treated with CQ was significantly decreased (*P* < 0.05) (**Figure 6B**). These results indicate that autophagy plays a role in regulating LA-induced inflammation *in vitro*.

## Discussion

Based on previous research, the role of autophagy varies in different tissues and physiological states (5). In this study, the results indicated that activation of autophagy in the liver or hepatocytes could reduce the gene expression of proinflammatory factors, such as TNFα and IL1β. Similarly, the findings of Zheng et al. and White et al. suggested that moderate activation of autophagy can alleviate inflammation in mammals and zebrafish (19, 20). The results of the present study showed that inhibition of autophagy upregulated the gene expression of proinflammatory factors *in vivo* and *in vitro*, which indicated that inhibition of basic autophagy could aggravate inflammation. The results were consistent with the findings of studies in mammals (21, 22). The role of autophagy in regulating the inflammation of large yellow croaker was preliminarily proven, although the molecular mechanism and signal pathway network underlying autophagy-related regulation of the inflammatory response in large yellow croaker needs to be further studied.

In this study, the role of autophagy in regulating linoleic acid-induced inflammation in the liver of large yellow croaker *in vivo* and *in vitro* was also studied. The results showed that activation of autophagy reduces LA-induced inflammatory cytokine gene expression *in vivo* and *in vitro* while inhibition of autophagy obtained the opposite results. These results suggested that autophagy is involved in the mitigation of the LA-induced inflammatory response, which is similar to the results of n-3 PUFAs (23–25). Previous studies have demonstrated that DHA activates autophagy to alleviate lipid toxicity in mammals, ursolic acid activates autophagy through the AMPK pathway to alleviate cell death and mitochondrial damage caused by PA, and n-3 PUFAs activate autophagy to inhibit inflammation through the SIRT1 pathway (23–25). Consequently, autophagy could alleviate LA-induced inflammation and we hypothesized that the molecular mechanism of autophagy alleviating the LA-induced inflammatory response might be related to the antioxidant system in the liver of large yellow croaker (13). Because previous studies in our laboratory have shown that autophagy could relieve oxidative stress and enhance antioxidant capacity (13). However, inhibiting autophagy can damage the antioxidant capacity (13). To the best of our knowledge, the role of autophagy in regulating LA-induced inflammation has not been previously studied in any species. In this study, autophagy was shown to alleviate LA-induced inflammation *in vivo* and *in vitro* for the first time.

In conclusion, our study reported for the first time that autophagy could regulate inflammation and alleviate LA-induced inflammation in the liver of large yellow croaker *in vivo* and *in vitro*. The present study identifies important pathways for alleviating inflammation, and these insights may offer great benefits to the aquaculture industry and for human health.

## Data Availability Statement

The original contributions presented in the study are included in the article/**Supplementary Material**. Further inquiries can be directed to the corresponding author.

## Ethics Statement

The animal study was reviewed and approved by Institutional Animal Care and Use Committee of Ocean University of China. The study was conducted according to the guidelines of the Declaration of Helsinki, and approved by the Institutional Review Board of the State Council (Chinese order no. 676, revised 1 March, 2017).

## Author Contributions

BY, RJ, XL, WF, QCC, QC, WX, and KM designed the research, conducted the experiments, analyzed the data, and wrote the manuscript. QA reviewed the manuscript and acquired funding. All authors contributed to the article and approved the submitted version.

## Funding

This work was supported by the National Science Fund for Distinguished Young Scholars of China (grant number: 31525024), the Key Program of National Natural Science Foundation of China (grant number: 31830103), the Agriculture Research System of China (grant number: CARS-47-11), the Scientific and Technological Innovation of Blue Granary (grant no. 2018YFD0900402) and the Ten-thousand Talents Program (grant no. 2018-29).

## Conflict of Interest

The authors declare that the research was conducted in the absence of any commercial or financial relationships that could be construed as a potential conflict of interest.
