# Supplementary material for: Activation of Autophagy Relieves Linoleic Acid-Induced Inflammation in Large Yellow Croaker (Larimichthys crocea)
Source: Front Immunol. 2021 Jun 30;12:649385. doi: 10.3389/fimmu.2021.649385 (PMC8279755; doi:10.3389/fimmu.2021.649385)
Supplement: Supplementary file 1 [file DataSheet_1.pdf]

Supplementary tables and figures:

Table 1. Formulation and proximate analysis of the experimental diets (% dry matter) [25]

Ingredients	Diets	
	FO[a]	SO[b]
Casein	36.80	36.80
Gelatin	9.20	9.20
Dextrin	28.00	28.00
Microcrystalline Cellulose	3.00	3.00
α-Starch	6.35	6.35
Mineral premix [c]	2.00	2.00
Vitamin premix [d]	2.00	2.00
Attractant [e]	0.30	0.30
Mould inhibitor [f]	0.10	0.10
Lecithin	2	2
Antioxidant	0.05	0.05
Choline chloride	0.20	0.20
Fish oil	10.00	0
Soybean oil	0	10.00
Total	100.00	100.00
Chemical proximate composition (%dry matter)		
Crude protein	43.38	42.57
Crude lipid	11.23	11.32

All those ingredients were supplied by Great Seven Biotechnology Co.,Ltd, China.

[a] FO: Fish oil group

[b] SO: soybean oil replacing fish oil at 100%

[c] Mineral premix (mg or g kg^{-1} diet): $CuSO_4 \cdot 5H_2O$ 10 mg; Na_2SeO_3 (1%) 25 mg; $ZnSO_4 \cdot H_2O$, 50 mg; $CoCl_2 \cdot 6H_2O$ (1%) 50 mg; $MnSO_4 \cdot H_2O$ 60 mg; $FeSO_4 \cdot H_2O$ 80 mg $Ca(IO_3)_2$ 180 mg; $MgSO_4 \cdot 7H_2O$ 1200 mg; zeolite 18.35 g

[d] Vitamin premix (mg or g kg-1diet): vitamin D 5 mg; vitamin K 10 mg; vitamin B12 10 mg; vitamin B6 20 mg; folic acid 20 mg; vitamin B1 25 mg; vitamin A 32 mg; vitamin B2 45 mg; pantothenic acid 60 mg; biotin 60 mg; niacin acid 200 mg; α-tocopherol 240 mg; inositol 800 mg; ascorbic acid 2000 mg; microcrystalline cellulose 16.47 g

[e] Phagostimulant: Glycine/ Betaine = 1:3

[f] Preservative: Fumarate/ Calcium pnpionabe = 1:1

Table 2. Fatty acids composition in the experimental diets (% total fatty acid) [25]

Fatty acid	Diets	
	FO	SO
14:0	5.44	0.60
16:0	21.30	13.00
18:0	4.54	4.54
20:0	0.61	0.39
ΣSFA	31.89	18.53
16:1n-7	5.85	0.29
18:1n-9	16.56	26.44
18:1n-7	3.16	—
20:1n-9	2.53	0.41
ΣMUFA	28.10	27.14
18:2n-6	10.16	47.92
20:4n-6	0.83	—
Σn-6PUFA	10.99	47.92
18:3n-3	2.04	4.76
20:5n-3(EPA)	6.40	0.16
22:6n-3(DHA)	8.64	—
Σn-3PUFA	17.08	4.92
n-3/n-6PUFA	1.55	0.10
Σn-3LC-PUFA	15.04	0.16

DHA/EPA	1.35	—

The low-level fatty acids are not list on. "—" means not detected. SFA, saturated fatty acids; MUFA, mono-unsaturated fatty acids; n-6 PUFA, n-6 poly-unsaturated fatty acids; n-3 PUFA, n-3 poly-unsaturated fatty acids; LC-PUFA, long chain-polyunsaturated fatty acids.

Table 3. Effect of dietary lipids on liver fatty acid composition (% total fatty acid) [25]

Fatty acid	Liver	
	FO	SO
14:0	2.84	0.85
16:0	24.00	10.56
18:0	8.00	8.03
20:0	0.24	0.23
ΣSFA	35.09	19.68
16:1n-7	9.47	3.02
18:1n-9	28.46	34.79
18:1n-7	2.74	1.62
20:1n-9	2.00	0.80
ΣMUFA	42.67	40.23
18:2n-6	7.16	34.25
20:4n-6	0.47	0.13
Σn-6PUFA	7.63	34.39
18:3n-3	1.18	2.43
20:5n-3(EPA)	2.03	0.14
22:6n-3(DHA)	2.89	0.16
Σn-3PUFA	6.10	2.86
n-3/n-6PUFA	0.81	0.08
Σn-3LC-PUFA	4.92	0.43

DHA/EPA	1.41	1.91

The low-level fatty acids are not list on. SFA, saturated fatty acids; MUFA, mono-unsaturated fatty acids; n-6 PUFA, n-6 poly-unsaturated fatty acids; n-3 PUFA, n-3 poly-unsaturated fatty acids; LC-PUFA, long chain-polyunsaturated fatty acids.
